# Influenza Among Young Children in Bangladesh: Clinical Characteristics and Outcomes From a Randomized Clinical Trial

**DOI:** 10.1093/cid/cix674

**Published:** 2017-10-06

**Authors:** Elizabeth Rotrosen, K Zaman, Jodi Feser, Justin R Ortiz, Doli Goswami, Amina Tahia Sharmeen, Mustafizur Rahman, Kristen D C Lewis, Md Ziaur Rahman, Burc Barin, W Abdullah Brooks, Kathleen M Neuzil

**Affiliations:** 1 Center for Vaccine Development, University of Maryland, Baltimore; 2 International Centre for Diarrhoeal Disease Research, Bangladesh, Dhaka; 3 PATH; 4 Departments of Medicine and Global Health, University of Washington, Seattle; 5 EMMES Corporation, Rockville; 6 Department of International Health, Johns Hopkins University, Baltimore, Maryland

**Keywords:** pediatrics, influenza, live attenuated influenza vaccine, influenza-like illness, developing country

## Abstract

**Background:**

Influenza causes substantial morbidity in children worldwide, although influenza vaccine is seldom used in low-resource settings. More information on the clinical presentation of influenza and the efficacy of vaccine is needed to inform policy.

**Methods:**

In 2013 we conducted a randomized, placebo-controlled clinical trial of live attenuated influenza vaccine (LAIV) in children aged 24–59 months in Bangladesh (N = 1761). If participants met prespecified specimen collection criteria, we collected nasopharyngeal washes for testing by singleplex reverse-transcription polymerase chain reaction (RT-PCR) for laboratory-confirmed influenza virus infection (LCI). A panel of RT-PCR assays was used to detect noninfluenza respiratory viruses. Primary efficacy results have been reported. In this analysis of prespecified and post hoc objectives from the trial, we compared signs and symptoms between LCI and non-LCI cases and estimated the efficacy of LAIV against moderate-to-severe LCI and other prespecified non-LCI clinical outcomes including all-cause pneumonia and acute otitis media.

**Results:**

The most common signs and symptoms of LCI were fever, cough, and runny nose. The combination of subjective fever and cough had a 63% sensitivity for LCI. The combination of measured fever, cough, and runny nose was most specific (90%) but had low sensitivity (32%) for LCI. The efficacy of LAIV against vaccine-strain moderate-to-severe LCI was 56.7% (95% confidence interval, 9.5%–79.2%). No statistically significant vaccine efficacy was found against the non-laboratory-confirmed clinical outcomes.

**Conclusions:**

It was not possible to distinguish LCI from noninfluenza viral infections on clinical evaluations alone in this population of Bangladeshi children. LAIV was efficacious against moderate-to-severe LCI.

**Clinical Trials Registration:**

NCT01797029.

## Teaser

In a live attenuated influenza vaccine (LAIV) trial among young Bangladeshi children, we found no presenting combinations of clinical signs and/or symptoms that could distinguish influenza virus infection from other acute respiratory infections. LAIV was efficacious against moderate-to-severe influenza illness.

Influenza is a common respiratory infection that occurs globally. Influenza incidence and severity differ from year to year, in part due to the circulation of differing viral strains. While the characteristics and prevention of influenza virus infection have been studied extensively in many parts of the world, influenza and influenza vaccines are not as well studied in low-resource tropical regions [1, 2].

In 2013, we conducted a randomized, placebo-controlled clinical trial to determine the efficacy of live attenuated influenza vaccine (LAIV) against laboratory-confirmed influenza illness (LCI) in children 24–59 months of age at field sites in urban and rural Bangladesh [3]. In that study, influenza attack rates in the placebo group were high, at 24.5% for all strains and 15.8% for vaccine-matched strains, with efficacy of 41.0% (95% confidence interval [CI], 28.0%–51.6%) and 57.5% (95% CI, 43.6%–68.0%), respectively. That study, and prior years of influenza surveillance in Bangladesh, has shown that influenza circulates during most months of the year [3] and that this circulation may overlap with other common respiratory viruses.

Here, we further examine the data from that 2013 efficacy trial to describe the clinical presentation of LCI in Bangladeshi children, the circulation of other viral etiologies of acute respiratory infections, and the effect of LAIV on clinical outcomes not previously reported. We compare clinical features of LCI and non-LCI illnesses to determine whether certain clinical signs and symptoms can accurately predict LCI in young Bangladeshi children. We also evaluate the efficacy of LAIV against additional outcomes.

## PATIENTS AND METHODS

The study was a phase 3, randomized, double-blind, placebo-controlled trial that evaluated the efficacy of LAIV in reducing the rates of symptomatic LCI in Bangladeshi children <5 years of age who received LAIV compared to placebo. Written informed consent was obtained from the parents/guardians of all participants. The study was approved by the ethical review committees of the International Center for Diarrhoeal Disease Research, Bangladesh (icddr,b) and by the Western Institutional Review Board (Puyallup, Washington). The study was conducted in compliance with the Declaration of Helsinki (2008) and complied with Good Clinical Practice guidelines. The study is registered on ClinicalTrials.gov (NCT01797029) [3].

Detailed study methods and primary results have been previously published [3]. In brief, 1761 participants aged 24–59 months received LAIV or placebo between February and April 2013; participants were monitored prospectively for predefined study outcomes through December 2013. The study was conducted at 2 sites: Kamalapur, an urban location, and rural Matlab. During weekly home visits, field workers (FWs) identified participants who met evaluation criteria, defined as either 1 category A finding, or, if absent, at least 2 category B findings. Category A findings included fever (≥38°C, axillary), respiratory rate ≥40 breaths/minute, danger signs (chest indrawing, lethargy, cyanosis, inability to drink, convulsions), labored breathing, noisy breathing, ear pain, and ear discharge. Category B findings included subjective fever, cough, rhinorrhea, sore throat, myalgia/arthralgia, chills, headache, irritability/decreased activity, and vomiting. Evaluation criteria were intentionally made to be broad to ensure comprehensive capture of LCI cases.

Participants who met protocol-defined evaluation criteria were evaluated by a study physician to determine if they met protocol-defined specimen collection criteria. Specimen collection criteria were defined as participants who either (1) presented to the clinic with influenza-like illness (ILI), defined in the study as having ≥2 of the following on the same day: measured temperature ≥37.5°C (considered an objective fever), cough, sore throat, runny nose, and congestion; or (2) met clinical illness criteria (including pneumonia, acute otitis media, meningitis, and sepsis), as defined in the study protocol [3, 4].

Participants who met specimen collection criteria had a nasopharyngeal wash (NPW) specimen collected and tested for influenza virus using reverse-transcription polymerase chain reaction (RT-PCR). These specimens were also tested for a panel of respiratory viruses (adenovirus, human parainfluenza viruses 1, 2, and 3 [HPIV-1, -2, -3, respectively], respiratory syncytial virus [RSV], and human metapneumovirus [hMPV]) using an RT-PCR assay [5]. Blood cultures were collected if clinically indicated. RT-PCR was performed on NPW specimens at the icddr,b virology laboratory and influenza-positive specimens were cultured to isolate virus for antigenic characterization at the US Centers for Disease Control and Prevention (CDC).

Subjects in Matlab were referred to Matlab Hospital; those in Kamalapur were referred to icddr,b Dhaka Hospital, or Monowara Hospital for care. Each of these hospitals has an established system to alert study physicians of hospitalizations. Children who self-referred to any other hospitals were identified by FWs at home visits. Study physicians evaluated all hospitalized subjects for safety outcomes; collected clinical data, determined if subjects met specimen collection criteria, and collected NPW specimens when appropriate. Moderate-to-severe LCI was defined as having an NPW specimen that was RT-PCR–positive for influenza as well as a measured temperature of at least 39°C and/or a physician diagnosis of any illness that met clinical illness criteria, as above.

### Statistical Considerations

The primary study efficacy and safety objectives have been previously reported [3]. Additional secondary objectives were (1) to describe the clinical characteristics of LCI in the study population and (2) to describe the viral etiologies of influenza-like illness in the study population. A prespecified exploratory objective was to estimate the efficacy of LAIV in reducing rates of moderate-to-severe LCI virus infection. In this analysis, we investigated these prespecified secondary and exploratory objectives. As a post hoc exploratory analysis, we also compared the efficacy of LAIV against clinical illnesses, based on physician diagnoses, regardless of laboratory confirmation, between the vaccinated and unvaccinated treatment groups. Finally, we evaluated the predictive power of certain clinical signs and symptoms for LCI. For our analyses, we considered signs and symptoms that occurred in at least 5% of the population.

All analyses were limited to the first episode of illness that occurred ≥8 days after vaccination for each subject. For the purposes of evaluating vaccine efficacy against moderate-to-severe LCI, the most severe LCI episode was identified for each participant and included in the efficacy analysis. Confirmed vaccine-type influenza illnesses within 14 days following vaccination were not considered to satisfy the LCI endpoints. The vaccine efficacy analyses against moderate-to-severe LCI and clinical illnesses were based on the entire cohort of 1761 participants whereas the remaining analyses focusing on clinic visit assessments were based on the subset of 1185 cases who had at least 1 clinic visit for NPW collection. Vaccine efficacy, expressed as a percentage, was defined as 1 minus the relative rate of illness in the LAIV group compared with that in the placebo group. Efficacy with 95% CIs was computed with a binomial distribution of LAIV cases. Fisher exact test was used for comparisons of clinical signs and symptoms between treatment groups among LCI cases, and between LCI and noninfluenza groups. Statistical analyses were performed using SAS version 9.3.

## RESULTS

**Figure 1.**
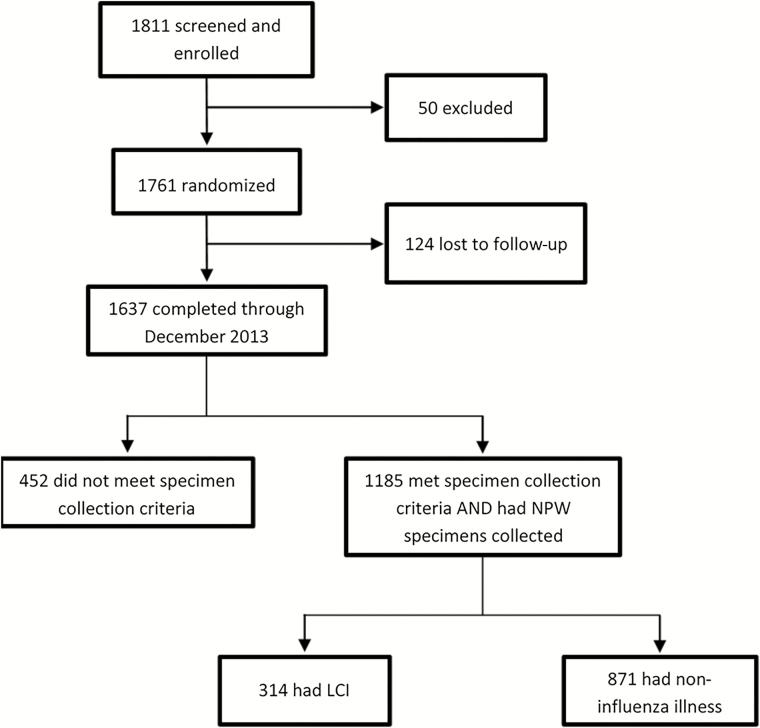
The CONSORT (Consolidated Standards for Reporting Trials) flow diagram for the study population through December 2013. Abbreviations: LCI, laboratory-confirmed influenza; NPW, nasopharyngeal wash.

Between February and April of 2013, we screened 1811 possible participants, 1761 (97%) of whom met inclusion criteria, were enrolled in the trial, and randomly assigned in a 2:1 ratio to receive LAIV or placebo. Of those enrolled and randomized, 1637 (93%) completed the study through December 2013 and 124 (7%) were lost to follow-up. Overall, 1185 (72%) of the participants who completed the study met criteria to be evaluated by study physicians at least 8 days postvaccination and had NPW specimens collected (Figure 1).

Participants who met specimen collection criteria and had an NPW specimen collected were demographically similar to the full study population (Table 1). Of the 1185 participants in this subpopulation, 314 (26.5%) had LCI, of whom 146 also had a noninfluenza viral illness; 467 (39.4%) had a noninfluenza viral illness only (ie, no LCI); and 404 (34.1%) had no RT-PCR–confirmed illness. Noninfluenza viruses were detected in 613 of the participants with NPW specimens collected and included adenovirus (24.1%), RSV (8.4%), hMPV (17.1%), HPIV-1 (6.6%), HPIV-2 (1.7%), and HPIV-3 (8.4%).

**Table 1.** Demographics and Medical Characteristics of Participants Who Completed Follow-up Through December 2013

Characteristic	Full Study Population (N = 1761)	Per-Protocol Population Meeting Specimen Collection Criteria (n = 1185)
Mean age, mo (range)	42.4 (24–59)	41.6 (24–59)
Age group		
2 to <3 y	544 (30.9)	401 (33.8)
3 to <4 y	537 (30.5)	357 (30.1)
4 to <5 y	680 (38.6)	427 (36.0)
Sex		
Male	866 (49.2)	583 (49.2)
Female	895 (50.8)	602 (50.8)
Site		
Kamalapur	1200 (68.1)	679 (57.3)
Matlab	561 (31.9)	506 (42.7)
Medical history of hospitalization with asthma or wheezing illness	78 (4.4)	57 (4.8)
Medical history of receiving treatment for asthma or wheezing illness	376 (21.4)	238 (20.0)

Data are presented as No. (%) unless otherwise indicated.

Among all children with an NPW specimen tested, we compared clinical findings between children with and without LCI. Among patients with LCI, fever (measured or subjective), cough, and runny nose were the most common findings, with each occurring in at least 65% of the population (Table 2). The frequency of the signs and symptoms reported in Table 2 did not differ significantly between treatment groups. Of these individual common symptoms, only fever (measured or subjective) occurred with significantly higher frequency in children with LCI than in children without LCI. 20 children were hospitalized during the first study follow-up period (through December 2013). Of those who were hospitalized, 8 met specimen collection criteria and 7 had NPW specimens collected. Of those 7, 1 child had LCI, 1 had adenovirus, and 1 had RSV; all others were negative for all viruses. LAIV did not have a statistically significant effect on hospitalizations (*P* = 1.000). Subjective fever was more sensitive (90.1% compared to 68.8%), but less specific (34.7% compared to 64.5%) than measured fever for LCI. Sensitivities, specificities, and positive and negative predictive values (PPVs and NPVs, respectively) for signs and symptoms’ association with and without LCI are reported in Supplementary Table 1. When considering combinations of symptoms, the presence of subjective fever and cough had the highest sensitivity for LCI (63.1%), but a relatively low specificity (59.7%) and PPV (36.1%). The combination of measured fever, runny nose, and cough was most specific (89.7%), but had low sensitivity (32.2%) for LCI. The combination of measured fever and cough had a sensitivity of 43.3%, specificity of 86.6%, PPV of 53.8%, and NPV of 80.9%.

**Table 2.** Proportion of Subjects With Documented Signs and Symptoms at Clinic Visits, March–December 2013^a^

Sign or Symptom^b^	Total (N = 1185)		Influenza Positive (n = 314)^c^		Influenza Negative (n = 871)^d^		Fisher Exact 2-Tailed Test
	No. (%)	(Exact 95% CI)	No. (%)	(Exact 95% CI)	No. (%)	(Exact 95% CI)	*P* Value
Fever (measured ≥38°C)	525 (44.3)	(41.5–47.2)	216 (68.8)	(63.3–73.9)	309 (35.5)	(32.3–38.8)	<.001
Fever (subjective)	852 (71.9)	(69.2–74.4)	283 (90.1)	(86.3–93.2)	569 (65.3)	(62.1–68.5)	<.001
Runny nose	832 (70.2)	(67.5–72.8)	217 (69.1)	(63.7–74.2)	615 (70.6)	(67.5–73.6)	.615
Cough	852 (71.9)	(69.2–74.4)	227 (72.3)	(67.0–77.2)	625 (71.8)	(68.6–74.7)	.884
Sore throat	54 (4.6)	(3.4–5.9)	9 (2.9)	(1.3–5.4)	45 (5.2)	(3.8–6.9)	.114
Fever (measured ≥38°C) and runny nose	242 (20.4)	(18.2–22.8)	122 (38.9)	(33.4–44.5)	120 (13.8)	(11.6–16.2)	<.001
Fever (measured ≥38°C) and cough	253 (21.4)	(19.0–23.8)	136 (43.3)	(37.8–49.0)	117 (13.4)	(11.2–15.9)	<.001
Fever (measured ≥38°C) and runny nose and cough	191 (16.1)	(14.1–18.3)	101 (32.2)	(27.0–37.6)	90 (10.3)	(8.4–12.5)	<.001
Fever (subjective) and runny nose	526 (44.4)	(41.5–47.3)	186 (59.2)	(53.6–64.7)	340 (39.0)	(35.8–42.4)	<.001
Fever (subjective) and cough	549 (46.3)	(43.5–49.2)	198 (63.1)	(57.5–68.4)	351 (40.3)	(37.0–43.6)	<.001
Fever (subjective) and runny nose and cough	453 (38.2)	(35.5–41.1)	160 (51.0)	(45.3–56.6)	293 (33.6)	(30.5–36.9)	<.001

Abbreviation: CI, confidence interval.

^a^Any sign or symptom with prevalence of at least 5% in either population.

^b^Each sign or symptom can be reported at the first clinic visit where influenza was documented (influenza positive) or was not documented (influenza negative), after 7 days postvaccination.

^c^Number of subjects with at least 1 documented episode of influenza.

^d^Number of subjects without documented influenza at any clinic visit.

The efficacy of LAIV against moderate-to-severe LCI and clinical outcomes regardless of laboratory confirmation of influenza is reported in Table 3. None of the differences for non-laboratory-confirmed outcomes were statistically significant between the treatment groups. The efficacy of LAIV against moderate-to-severe LCI caused by vaccine-matched strains was 56.7% (95% CI, 9.5%–79.2%) and caused by any influenza virus strain was 48.0% (95% CI, 10.8%–69.7%) (Table 3). In this population, all-cause, clinically diagnosed pneumonia was more common in the placebo group (6%) compared with to the LAIV group (4.3%). The resulting efficacy estimation was 28.6% (95% CI, –8.8% to 53.1%). With regards to acute otitis media (AOM), there were 70 cases in the LAIV group (6%) and 25 cases (4.3%) in the placebo group, corresponding to an efficacy of –40% (95% CI, –118.6% to 10.4%).

**Table 3.** Proportion of Subjects With Preselected Clinical Outcomes by Treatment Group, March–December 2013

Clinical Diagnosis^a,b,c^	Total (N = 1761)^d^		LAIV (n = 1174)^d^		Placebo (n = 587)^d^		Risk Difference (LAIV-Placebo)			Efficacy, % (95% CI)
	No. (%)	(Exact 95% CI)	No. (%)	(Exact 95% CI)	No. (%)	(Exact 95% CI)	Difference, %	(95% CI)	Fisher Exact 2-Tailed Test	
Upper respiratory infection	379 (21.5)	(19.6–23.5)	251 (21.4)	(19.1–23.8)	128 (21.8)	(18.5–25.4)	–0.4	(–4.5 to 3.7)	0.854	2.0 (–18.4 to 18.8)
Pneumonia^e^	85 (4.8)	(3.9–5.9)	50 (4.3)	(3.2–5.6)	35 (6.0)	(4.2–8.2)	–1.7	(–3.9 to .5)	0.126	28.6 (–8.8 to 53.1)
Severe pneumonia^f^	6 (0.3)	(.1–.7)	2 (0.2)	(.0–.6)	4 (0.7)	(. 2–1.7)	–0.5	(–1.2 to 0.2)	0.100	75.0 (–36.1 to 95.4)
Very severe pneumonia^g^	0 (0.0)	(.0–.2)	0 (0.0)	(.0–.3)	0 (0.0)	(.0–.6)	0	…	…	…
Acute otitis media^h^	95 (5.4)	(4.4–6.6)	70 (6.0)	(4.7–7.5)	25 (4.3)	(2.8–6.2)	1.7	(–.4 to 3.8)	0.147	–40.0 (–118.6 to 10.4)
Asthma/reactive airway disease	49 (2.8)	(2.1–3.7)	32 (2.7)	(1.9–3.8)	17 (2.9)	(1.7–4.6)	–0.2	(–1.8 to 1.5)	0.878	5.9 (–68.1 to 47.3)
Moderate to severe influenza (any strain)	51 (2.9)	(2.2–3.8)	26 (2.2)	(1.5–3.2)	25 (4.3)	(2.8–6.2)	–2.0	(–3.9 to–.2)	0.023	48.0 (10.8–69.7)
Moderate to severe influenza (vaccine matched)	28 (1.6)	(1.1–2.3)	13 (1.1)	(.6–1.9)	15 (2.6)	(1.4–4.2)	–1.4	(–2.9 to –.0)	0.026	56.7 (9.5–79.2)

Abbreviation: CI, confidence interval; LAIV, live attenuated influenza vaccine.

^a^Each clinical diagnosis can be reported at any or all clinic visits after 7 days postvaccination, but can only be counted once per subject.

^b^Diagnoses are according to physician diagnosis, with the exception of pneumonia, then run according to per-protocol definitions.

^c^Clinical diagnoses with a prevalence of at least 2% in either population were included in the table with the exceptions of severe pneumonia and very severe pneumonia.

^d^Number of subjects in the full study population.

^e^Pneumonia was defined as (1) age-specific tachypnea (≥ 40 breaths/minute if age 24–59 months), AND (2) crepitations on auscultation.

^f^Presence of pneumonia and chest indrawing.

^g^Presence of pneumonia and at least 1 danger sign (central cyanosis, severe respiratory distress, convulsions, altered mental status).

^h^Acute otitis media defined as injection (rubor) of the tympanic membrane and/or reduced movement on insufflations. Suppurative otitis media will have purulent fluid from the ear.

**Figure 2.**
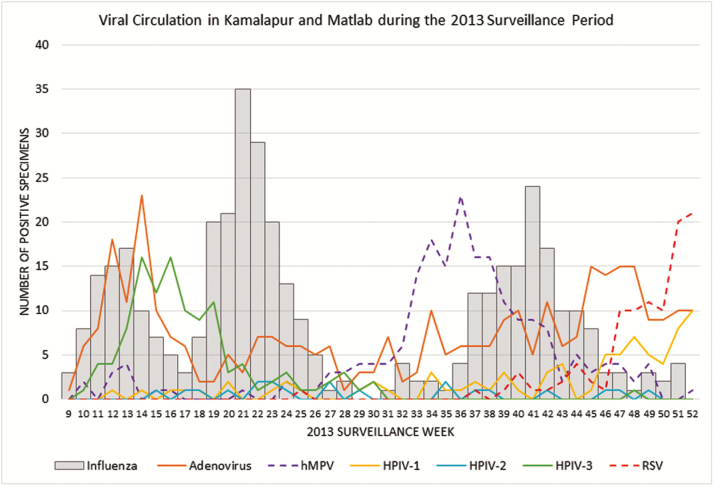
Number of specimens that tested positive for selected respiratory viruses by study surveillance week. Gray vertical bars indicate number of cases of influenza by surveillance week; other respiratory viruses are indicated as colored lines, as shown in the figure key. Surveillance week correlates to calendar week. Abbreviations: hMPV, human metapneumovirus; HPIV, human parainfluenza virus; RSV, respiratory syncytial virus.

Figure 2 shows the circulation patterns of the viruses isolated in NPW specimens during the trial surveillance period. Influenza was biphasic and adenovirus showed a similar temporal pattern. hMPV circulated predominantly in the period between influenza outbreaks. RSV circulation continued to increase from a low baseline toward the end of our study in December, suggesting that the study surveillance period, which excluded the January through March time period, may not have captured the RSV peak or the entirety of the RSV season.

## DISCUSSION

Our original study on the efficacy of LAIV in young children in urban and rural Bangladesh provided an opportunity to better understand the clinical presentation of LCI in this population. In this cohort of 24- to 59-month-old children, it was not possible to clinically differentiate LCI from other noninfluenza illnesses. Fever, both measured and subjective, cough, and runny nose were the most common presentations of LCI, with each occurring in at least 65% of LCI illnesses. These symptoms were also common in noninfluenza illnesses and only fever was significantly associated with LCI. When combinations of fever with additional findings were assessed, sensitivity decreased while specificity increased. The combined findings of measured fever, cough, and runny nose had the highest specificity (89.7%) at the expense of sensitivity (32.2%). The PPVs of subjective fever (33.2%), measured fever, cough, and runny nose (52.9%), and subjective fever and runny nose (35.4%) were relatively low, suggesting that none of these combinations of signs and symptoms is a particularly good indicator of LCI. The combination of measured fever and cough was not highly predictive either (PPV, 53.8%).

Our findings must be interpreted in the context of our evaluation and specimen collection criteria. However, these findings from a tropical, low-resource setting are similar to those from temperate, high-resource settings [6–9], and demonstrate the challenges to diagnosing influenza with clinical criteria alone. For example, Ohmit and Monto conducted a retrospective analysis of children 1–12 years old who participated in a randomized, double-blind, placebo-controlled trial of oral oseltamivir treatment for influenza, conducted across 70 sites in the United States and 10 sites in Canada [10, 11]. They found a PPV of 64% (95% CI, 58%–71%) for LCI in 221 children <5 years old who presented with a combination of measured fever (≥38.2°C, axial) and cough. This study, however, tested for and excluded all children with RSV, limiting the comparability to our results.

Our study adds important information on the clinical presentation of influenza in tropical regions, where data are limited. The World Health Organization (WHO) defines influenza-like illness as “an acute respiratory infection with measured fever of ≥38.0°C and cough” [4]. This definition is used for annual influenza surveillance reported by the WHO. From our data, it is clear that although influenza circulates in Bangladesh with notable differences in circulation patterns as compared to temperate regions, including the United States, the major signs and symptoms of influenza illness are similar to those in children in the United States.

Many noninfluenza, viral, and bacterial infections commonly occur with overlapping seasonality with influenza. As we, and others, have shown, acute respiratory infections, caused by other viruses and bacteria, can be difficult to distinguish clinically from influenza [12–14]. This supports the importance of diagnostic testing, which is often not available in low-resource settings. Of the noninfluenza viruses detected during our surveillance period, adenovirus had the highest attack rate, followed by hMPV, HPIV-3, RSV, HPIV-1, and HPIV-2. However, we suspect the RSV attack rate was underestimated, due to the timing of our surveillance, which missed the first quarter of the calendar year. LAIV receipt did not confer protection against noninfluenza viruses.

In low-resource settings with many competing health priorities, efficacy against any-severity influenza may not be sufficient to drive a policy decision. Therefore, we also evaluated the efficacy of LAIV against additional clinical outcomes of public health importance. LAIV prevented moderate-to-severe influenza, with an efficacy of 48.0% (95% CI, 10.8%–69.7%), against all strains of influenza and 56.7% (95% CI, 9.5%–79.2%) against vaccine-matched strains. These estimates are similar to those reported in the original publication for any severity influenza [3]. Pneumonia is a leading cause of early childhood death and disability in Bangladesh and globally. In the study population, the overall incidence of any or severe pneumonia was 48 per 1000 and 3 per 1000, respectively, by per-protocol definition through the first influenza season [15, 16]. We measured a nonsignificant vaccine efficacy against all-cause pneumonia (28.6% [95% CI, –8.8% to 53.1%). A significant effect of this magnitude would have a substantial impact at population level. Larger studies are urgently needed to understand the impact of influenza vaccines against all-cause pneumonia [17, 18].

AOM was also common, occurring in 5.4% of children in this population. A recent meta-analysis of 8 randomized controlled trials during 1996–2005, in which LAIV efficacy against influenza-associated AOM was a prespecified secondary endpoint, showed LAIV to be highly effective against AOM across multiple influenza seasons and geographic locations. In contrast, in this study, we were unable to show an effect against AOM [19]. While not statistically significant, AOM was more common in the LAIV group. A factor that may confound a direct comparison between other studies and our study in Bangladesh is that vaccine coverage for the major bacterial causes of AOM varies widely by country and is incomplete in low-resource settings. Pneumococcal conjugate vaccine (PCV) was first introduced in Bangladesh in March 2015. Therefore, during the study period, Bangladeshi children had not been administered PCV, and our results should be interpreted within that context [20, 21].

There were several limitations to this study. One is the strict criteria set for the collection of NPW specimens. While our evaluation criteria were broad, our specimen collection criteria were more restricted. It is therefore possible that our study overlooked cases of relatively mild influenza that did not meet the specimen collection criteria. Thus, the 314 cases with an episode of LCI may represent a minimal estimate of the incidence of influenza over the surveillance window. Another limitation of this study is that we only analyzed a single influenza season, and influenza circulation and attack rates may vary substantially from year to year.

The global burden of influenza remains high and falls predominantly on young children and other at-risk individuals [22, 23]. Moreover, without the availability of diagnostic tests, the true burden of influenza is most likely underestimated in many settings. In young children in Bangladesh, those with LCI presented in the clinic with fever, cough, and runny nose, although these symptoms were also common in other noninfluenza viral infections, so their presence was not a reliable indicator of influenza. LAIV was efficacious against moderate-to-severe LCI. We found no significant vaccine efficacy against all-cause pneumonia or acute otitis media, although our study was not powered for these outcomes. Larger studies are needed to demonstrate influenza vaccine efficacy against clinical endpoints of public health importance in low-resource settings.

## Supplementary Data

Supplementary materials are available at *Clinical Infectious Diseases* online. Consisting of data provided by the authors to benefit the reader, the posted materials are not copyedited and are the sole responsibility of the authors, so questions or comments should be addressed to the corresponding author.

## Supplementary Material

Supplementary_Appendix

## Notes

***Acknowledgments.*** We thank the families who participated in this trial as well as the research team in Bangladesh, especially the clinical and field teams at both sites. We thank Fatimah Dawood, US Centers for Disease Control and Prevention (Atlanta, Georgia) and Bilkis Ara Anjali (icddr,b, Dhaka, Bangladesh). We thank the Serum Institute of India (Pune) for donating the vaccine and placebo used in this study. We thank PATH (Seattle, Washington), and the teams at Clinogent (Hyderabad, India) and at EMMES (Rockville, Maryland) for support. We also thank Kristin Bedell at PATH for assistance with trial management.

***Financial support.*** This work was supported by funding from the Bill & Melinda Gates Foundation, which provides financial support to PATH’s Influenza Vaccine Project (OPP48805).

***Potential conflicts of interest.*** All authors: No reported conflicts of interest. All authors have submitted the ICMJE Form for Disclosure of Potential Conflicts of Interest. Conflicts that the editors consider relevant to the content of the manuscript have been disclosed.
