# Supplementary material for: Influenza Among Young Children in Bangladesh: Clinical Characteristics and Outcomes From a Randomized Clinical Trial
Source: Clin Infect Dis. 2017 Oct 6;65(11):1914–20. doi: 10.1093/cid/cix674 (PMC5850015; doi:10.1093/cid/cix674)
Supplement: Supplementary_Appendix [file cix674_suppl_supplementary_appendix.docx]

Supplementary Appendix

Supplementary Table 1

**Supplementary Table 1**

**Sensitivities, Specificities, Positive Predictive Values (PPV) and Negative Predictive Values (NPV) of**

**Statistically Significant Signs and Symptoms for LCI**

| SIGN or Symptom | Sensitivity (%) | Specificity (%) | PPV (%) | NPV (%) |
| --- | --- | --- | --- | --- |
| Measured Fever (≥38.0˚F) | 68.8 | 64.5 | 41.1 | 85.2 |
| Subjective Fever | 90.1 | 34.7 | 33.2 | 90.7 |
| Measured Fever AND Runny Nose | 38.9 | 86.2 | 50.4 | 79.6 |
| Measured Fever AND Cough | 43.3 | 86.6 | 53.8 | 80.9 |
| Measured Fever AND Runny Nose AND Cough | 32.2 | 89.7 | 52.9 | 78.6 |
| Subjective Fever AND Runny Nose | 59.2 | 61.0 | 35.4 | 80.6 |
| Subjective Fever AND Cough | 63.1 | 59.7 | 36.1 | 81.8 |
| Subjective Fever AND Runny Nose AND Cough | 51.0 | 66.4 | 35.3 | 79.0 |
